# Acceptance-based therapy: the potential to augment behavioral interventions in the treatment of type 2 diabetes

**DOI:** 10.1038/s41387-020-0106-9

**Published:** 2020-01-21

**Authors:** Michelle I. Cardel, Kathryn M. Ross, Meghan Butryn, W. Troy Donahoo, Abraham Eastman, Julia R. Dillard, Anna Grummon, Patrick Hopkins, Leah D. Whigham, David Janicke

**Affiliations:** 1 Department of Health Outcomes and Biomedical Informatics, University of Florida, Gainesville, FL 32611 USA; 2 Department of Clinical and Health Psychology, University of Florida, Gainesville, FL 32611 USA; 3 Department of Psychology, Drexel University, Gainesville, PA 19104 USA; 4 Department of Medicine, University of Florida, Gainesville, FL 32611 USA; 5 Department of Food Science and Human Nutrition, University of Florida, Gainesville, FL 32611 USA; 6 Center for Population and Development Studies, Harvard TH Chan School of Public Health, Gainesville, MA 02115 USA; 7 University of Texas El Paso, Houston, TX 79968 USA; 8 University of Texas Health Sciences Center School of Public Health, Houston, TX 77030 USA

**Keywords:** Weight management, Type 2 diabetes, Obesity

## Abstract

Diabetes is a complex and multifactorial disease affecting more than 415 million people worldwide. Excess adiposity and modifiable lifestyle factors, such as unhealthy dietary patterns and physical inactivity, can play a significant role in the development of type 2 diabetes. Interventions that implement changes to lifestyle behaviors, in addition to pharmacological treatment, may attenuate the development and worsening of diabetes. This narrative review delineates how standard behavioral interventions (SBTs), based in “first wave” behavioral therapies and “second wave” cognitive behavioral therapies, serve as the foundation of diabetes treatment by supporting effective lifestyle changes, including improving adherence to healthful behaviors, medication, and self-monitoring regimens. Moreover, “third wave” “acceptance-based therapies” (ABTs), which integrate techniques from acceptance and commitment therapy, are proposed as a potential novel treatment option for diabetes management. Further research and long-term, randomized controlled trials will clarify the feasibility, acceptability, and effectiveness of ABT for improving glucose control via enhancing medication adherence and promoting effective lifestyle changes in people with diabetes.

## Diabetes as a public health crisis

Diabetes is considered one of the largest epidemics in human history, affecting more than 415 million people worldwide^1^. In the U.S. alone, over 26 million people are currently living with diabetes^2^. Type 2 diabetes, a chronic condition where the body does not produce or use insulin well, accounts for over ninety percent of this disease burden^2^. Type 2 diabetes has a high mortality rate: individuals with diabetes have a fifty percent higher all-cause mortality rate compared to individuals without diabetes^3^. In addition, type 2 diabetes has a high rate of co-morbidity with other chronic diseases; for example, the leading cause of death for individuals with diabetes is coronary heart disease (CHD)^4^. In older populations, the overlap of these comorbidities leads to further complications, including hypertension and hyperlipidemia^5^. The high mortality and co-morbidity rate make the treatment of people with type 2 diabetes especially challenging^5^.

There are both environmental and genetic factors that contribute to the onset of type 2 diabetes; however, the unanticipated and drastic increase in individuals living with type 2 diabetes suggests the environmental component of this equation (e.g., an obesogenic environment, stress-related factors) is an important contributor to the present type 2 diabetes epidemic^3^. Related to these environmental exposures, obesity and modifiable lifestyle factors including excess calorie intake and physical inactivity have been established as key drivers of disease onset, progression, and prognosis^1^.

Despite the introduction of several new classes of type 2 diabetes medications onto the market over the last decade, there have not been observable improvements in achieving diabetes treatment targets at a population level^6^. Currently, at least 45% of patients with type 2 diabetes do not achieve adequate glycemic control (hemoglobin A1c <7%)^7^, and poor medication adherence has been well documented as a common contributor to not only poor glycemic control but also increased morbidity and mortality^7^. This lack of improvement indicates a lack of adherence to key elements of diabetes treatment, including behavioral and pharmacological intervention. Significant change is needed to enhance behavioral intervention, both to support improved medication adherence and to better help with lifestyle management. Thus, treatment must focus on both improving modifiable lifestyle factors including nutrition, physical activity, and medication adherence.

## Standard behavioral interventions are foundational for diabetes treatment

Along with improving medication adherence, standard behavioral therapy (SBT) interventions can support a healthy diet and regular engagement in physical activity, which are key in weight management and part of comprehensive diabetes treatment^3^. SBTs have demonstrated effectiveness for weight reduction^8^, producing reductions of 8–10% of initial weight in adults with overweight and obesity^9^, which helps both prevent and treat type 2 diabetes. The Diabetes Prevention Program (DPP), a landmark clinical trial conducted in adults with prediabetes, demonstrated that participation in a behavioral weight loss program was superior to pharmacological treatment for preventing type 2 diabetes^10^. In adults who have progressed to type 2 diabetes, weight losses of 5–10% have been associated with clinically significant improvements in glucose regulation and a reduction in patients’ need for diabetes medications^8^. Even smaller weight losses of 2–5% have been associated with improvements in fasting glucose and hemoglobin A1c^8^. Accordingly, current guidelines emphasize the importance of “comprehensive lifestyle interventions,” such as the SBT weight loss program utilized in the DPP in delaying progression to and managing type 2 diabetes^8,11^.

Initial behavioral interventions, based in early (or “first wave”) behavioral learning theories, were developed in the 1960s^12,13^. These early programs, which often did not include specific goals for caloric intake and physical activity, produced weight losses of ~4.5 kg^14,15^. Throughout the 1970s–1990s, outcomes for behavioral treatments were improved by lengthening the duration of intervention and integrating techniques drawn from self-regulation theory, social learning theory, and cognitive behavioral therapy^14,16^.

Modern SBT interventions (also referred to as “comprehensive lifestyle interventions”)^8^ are grounded in behaviorism, self-regulation theory, and social-cognitive theory^17–19^, and teach individuals to change eating and activity behaviors through use of effective goal setting, self-monitoring, problem solving, and stimulus control. Specifically, over the course of weekly, 60–90 min sessions delivered over a period of 4 to 6 months^9^, participants are taught how to craft short-term, achievable, and measurable goals; how to use regular self-monitoring (e.g., tracking weight, calorie intake, and physical activity) to assess progress towards these goals; and how to implement structured problem solving to make additional changes when goals are not met^17^. Training in stimulus control teaches participants how to make changes in their immediate environments in order to support the changes that they are making to their eating and physical activity habits.

Existing SBTs, such as the DPP, also integrate additional techniques adapted from cognitive-behavioral therapy (CBT). CBT is often referred to as a “second wave” therapy, building on “first wave” behavioral therapies by adding “cognitive” components focused on helping individuals identify and change maladaptive thinking patterns in order to modify feelings and behaviors^16,20^. In CBT-focused components of lifestyle interventions (e.g., the “Talk Back to Negative Thoughts session from the DPP)^21^, participants are taught how to engage in *cognitive restructuring*, or the ability to identify and challenge or “talk back to” irrational or maladaptive thoughts. Individuals are first taught to identify these thoughts and then to categorize them within known groups of “cognitive distortions.” For example, the category of *all-or-nothing thinking* includes interpretations of events at their extreme. In a diabetes management context, this is often observed when people say that they are “on” or “off” their diet. This thought is viewed as maladaptive as it can lead to a small lapse in behavior turning into a much larger lapse or even relapse^17^. As an illustration of this process, a person who overeats at a lunch with coworkers may have the thought that “I blew it.” This thought, in turn, may lead to feelings of frustration and anger, and additional overeating. Alternatively, a person who misses a planned workout may have the thought “I can’t do this,” and similar feelings of frustration and anger may lead to that person not attempting to exercise for the rest of the week. Once an individual has identified this thought as maladaptive and categorized it as *all-or-nothing thinking*, they would be taught to then implement strategies to challenge or re-frame that thought. For example, in the first scenario, a person could challenge the rationality of the thought and whether it was really true that overeating at one meal “ruins” his or her whole day. This person could then counter maladaptive thinking with an alternative thought that “Well, I ate more than I meant to at lunch just now, but it doesn’t mean the whole day is wasted—I can eat a little less at dinner to get back on track.”

While SBTs have demonstrated effectiveness for initial weight loss, long-term maintenance of weight loss remains a substantial challenge^22^. Most individuals begin regaining weight after the end of the initial intervention, gaining about 1/3 to 1/2 of lost weight within a year and gradually returning to baseline weight within 3–5 years^23^. These regains largely result from sub-optimal adherence to the behavior changes made during the initial intervention. This is important to note as the struggles for weight maintenance parallel the struggles that individuals with type 2 diabetes face when managing their blood glucose levels.

The provision of “extended care” maintenance programs, wherein participants are provided with continued support after the end of the initial intervention, have been shown to improve maintenance of initial weight losses^24,25^, but the improvements are modest. These interventions do not change the pattern of regain as much as they slow the rate of regain, such that participants in these interventions regain about 1.5 kg less at 24 months compared to participants receiving standard care^25^. To date, no SBT or SBT + CBT interventions have successfully changed the “check-mark” pattern of weight loss and regain demonstrated across the literature^22^, suggesting that newer approaches may be warranted.

## Acceptance-based treatments may serve as a novel treatment approach and address some of the limitations of SBT

One newer approach that has received considerable attention is acceptance-based interventions. Acceptance-based interventions build upon the behavioral skills used in traditional lifestyle programs by adding components derived from acceptance and commitment therapy (ACT). ACT has been described as a “third wave” behavioral therapy, following the “first wave” of initial behaviorism and the “second wave” of CBT^20^. As a larger therapeutic approach, ACT attempts to increase individuals’ “psychological flexibility,” or their ability to engage in behaviors consistent with their values despite uncomfortable thoughts, feelings, or cravings^26^. Core processes in ACT include the clarification of life “values” (e.g., *why* an individual would choose to engage in a target behavior) and building willingness to experience negative thoughts, emotions, and sensations in order to engage in behaviors consistent with those values.

In contrast to CBT approaches, ACT does not teach individuals to identify and change irrational or maladaptive thoughts; rather, the focus of ACT is on changing how an individual relates to these thoughts and feelings^26^. For example, someone who is trying to increase his or her physical activity may avoid exercising at a gym because this person worries about being judged by other patrons. In a CBT approach, this person would be encouraged to challenge or change that thought in order to go to the gym (e.g., considering “What is the worst that could happen?” “What are the odds that would actually happen?”). In contrast, in an ACT-based approach, this person would be taught acceptance skills that would encourage them to go to the gym to exercise even if they feel uncomfortable. The novelty and success of ACT-based interventions lie in its focus on self-regulation skills and its potential broad effectiveness across diverse populations^27^. Further, ACT has proven successful for treating individuals for diverse medical and behavioral issues including chronic pain, substance abuse, high-risk sexual behavior, anorexia among adolescent females, and depression^28–34^.

In adults with type 2 diabetes, only two studies have examined the efficacy of ACT relative to a control group for improving diabetes self-management behaviors and glycemic control. In one study, Gregg and colleagues randomized 81 adults with type 2 diabetes to one of two 7-h intervention workshops:^1^ diabetes education alone or^2^ combined diabetes education plus ACT^35^. Adults in the combined diabetes education plus ACT intervention were more likely to use ACT-based coping strategies, reported better diabetes self-care (including exercise, diet, and glucose monitoring), and showed a greater mean decrease in A1c. The ACT plus diabetes education condition also had a higher percentage of individuals with an A1c < 7.0 compared to adults in the diabetes education only group. Improvements in A1c from baseline to follow-up were mediated by participants’ use of acceptance-based coping and engagement in diabetes self-management behaviors. In the second study, Shayeghian and colleagues randomized 106 adults with type 2 diabetes to a diabetes education only condition or a diabetes education plus ACT intervention^36^. The diabetes education plus ACT intervention consisted of ten sessions over a three-month period, while adults in the diabetes education only group participated in a 2-h workshop on diabetes control^36^. At three months post-baseline, adults in the ACT intervention showed greater improvements in A1c and diabetes self-care activities (including exercise, diet, glucose monitoring, medication adherence, cigarette smoking, and foot care) relative to those in the control condition.

One promising direction for lifestyle modification is to take key elements of ACT and integrate them into evidence-based SBT weight management treatments (SBT + ACT). Researchers and practitioners are typically intentional in referring to this approach as “acceptance-based behavioral treatment” (ABT) rather than “acceptance and commitment therapy” (ACT) for two reasons: this language acknowledges the importance of retaining traditional behavioral components such as goal setting and self-regulation found in SBT, and also reflects that ABT interventions may not utilize all elements typically delivered in ACT. Traditional behavioral interventions (e.g., the DPP lifestyle intervention) have elements that are likely critical for maximizing changes in weight, eating behaviors, and physical activity, which can all significantly benefit weight loss and blood glucose control. Teaching ACT skills without these elements would likely compromise efficacy (and would be impractical, as ACT is at its core a behavioral treatment that incorporates behavioral targets). Thus, ABT often retains core behavioral components of traditional SBTs, including: use of structured goals for reducing caloric intake and increasing physical activity; regular self-monitoring of weight, eating, and physical activity; monitoring of weight and these self-monitoring records by a treatment provider to provide feedback, support, and to enhance supportive accountability^37^; and additional behavioral skills training such as goal setting, problem solving, and stimulus control. Cognitive restructuring and other CBT-based techniques designed to help individuals manage negative internal experiences are not retained, however, as ABT focuses on changing relationships with internal experiences rather than changing the experience itself. In the version of ABT that has been most extensively researched to date^38^, the ACT strategies that are integrated into the intervention are acceptance of uncomfortable states and emotions, increasing commitment to valued goals, and aligning your values with your actions. In addition, *cognitive defusion* is included but in a modified format, as other forms of internal experience, especially hedonic drive are thought to be as important as cognitive activity. For those familiar with ACT, it is important to note that other ACT strategies, such as *self as process* and *self as context*, receive less attention.

Two randomized controlled trials (RCTs) have demonstrated significantly greater weight losses for ABT compared to SBT^39,40^. The Mind Your Health Project, a 40-week RCT comparing ABT to SBT, demonstrated that ABT resulted in significantly higher weight loss than SBT at post-treatment (13.2 vs. 7.5%) and at 6-month follow-up (11.0 vs. 5.0%) in a post-hoc analysis when the intervention was administered by weight-control experts^41^. Similarly, the Mind Your Health II Project (MYH II), a larger and longer (1 year) RCT, demonstrated that ABT produced greater 12-month weight loss than did the SBT treatment (13.3 vs. 9.8%), again when conducted by experienced clinicians^39^. In the long-term post-treatment follow-up data from MYH II^40^, weight loss at 24-months (7.5 vs. 5.6%; *P* = 0.15) or at 36 months (4.7 vs. 3.3%; *P* = 0.31) did not significantly differ between ABT and SBT groups. However, among treatment completers who attained at least 10% body weight loss during the intervention, those receiving ABT were almost twice as likely to retain at least 10% body weight loss at two-year follow-up (31.6 vs. 17.1%; *P* = 0.04). Importantly, another study showed that ABT’s effectiveness for weight loss did not differ by race, sex, or education^27^.

Lillis and colleagues compared an ABT intervention to SBT in adults with both overweight/obesity and high internal disinhibition (the tendency to overeat in negative emotional states), given that these individuals have poorer outcomes in typical weight management trials^42^. While there was no difference in initial weight loss between the ABT and SBT groups^43^, ABT appeared superior on other dimensions: participants in the ABT group regained less weight at 24-month follow-up than those in the SBT condition (4.6 vs. 7.1 kg) and were significantly more likely to achieve at least 5% total body weight loss at 24-months (38 vs. 25%).

Other interesting variants of ABT are currently being explored. For example, a recent pilot study examined remote delivery of ABT to bariatric surgery patients that experienced postoperative weight regain, finding initial support for the feasibility, acceptability, and preliminary efficacy of this intervention^44^.

## Future research directions

Given the key roles of obesity and modifiable lifestyle factors (i.e., dietary intake, physical activity, adherence to blood glucose monitoring and medication regimens) in the prevention and management of type 2 diabetes, there remains great need to develop and disseminate effective acceptance-based behavioral interventions. To date, only two trials have investigated integration of ACT into diabetes management (e.g., blood glucose monitoring and mediation adherence)^35,36^. One of these studies taught ACT-based diabetes management strategies in a workshop^35^ and the other in a 10-session program^36^. While both of these studies showed improvements in exercise, diet, and glucose monitoring, additional research is needed. Specifically, no studies have attempted to fully integrate a comprehensive lifestyle management ABT program with ACT diabetes management described above, and no studies have examined the impact of ABT on glycemic control in adults with or at high risk for type 2 diabetes. Development of future acceptance-based behavioral interventions should also utilize measures that can help determine feasibility and scalability of this approach as this treatment currently requires highly trained practitioners for effective outcomes.

## Clinical implications

Behavioral intervention remains a key component of type 2 diabetes treatment and prevention; however, effective type 2 diabetes treatment often involves a broad approach with cooperation among many professionals^3^. Given the promising early evidence supporting ABT for diabetes management, if future research is consistent in showing positive outcomes with ABT, a strong argument can be made for including ABT delivered by experienced clinicians as part of a comprehensive team approach in the treatment of patients with prediabetes and diabetes. Further work also needs to be done on implementing ABT within primary care and community-based clinics, rather than in academic medical centers.

## Conclusion

The existing literature has demonstrated that ABT interventions are efficacious for producing weight loss in adults with overweight and obesity, and that ACT-based diabetes interventions hold early promise for improving glycemic outcomes. Future work is encouraged to investigate the long-term effectiveness of ABT outside of tightly-controlled trials conducted in academic medical centers and to determine whether combining ABT with diabetes management strategies improves diabetes-related outcomes in adults with or at high-risk for type 2 diabetes. Given the promise of ABT-based interventions, coupled with the growing burden of diabetes worldwide, additional funding and resources are warranted to evaluate the long-term effectiveness of ABT interventions for managing type 2 diabetes. ABT could provide a skillset that could be incorporated into existing programs (e.g., the DPP) and could be taught and used by a variety of providers, including psychologists, registered dietitians, certified diabetes educators, nurses, and physicians, community health workers, social workers, and others to improve lifestyle change and medication adherence among patients with diabetes.
